# Editorial: Disease-modifying antirheumatic drugs: Approaches and lessons learned from traditional medicine

**DOI:** 10.3389/fphar.2023.1135803

**Published:** 2023-02-03

**Authors:** Liyan Mei, Kaixin Gao, Xiaojuan He, Per-Johan Jakobsson, Runyue Huang

**Affiliations:** ^1^ The Second Affiliated Hospital, Guangzhou University of Chinese Medicine (Guangdong Provincial Hospital of Chinese Medicine), Guangzhou, China; ^2^ China Academy of Chinese Medical Sciences, Beijing, China; ^3^ Karolinska Institutet (KI), Solna, Sweden; ^4^ Guangdong-Hong Kong-Macau Joint Lab on Chinese Medicine and Immune Disease Research, Guangzhou University of Chinese Medicine, Guangzhou, China; ^5^ State Key Laboratory of Dampness Syndrome of Chinese Medicine, The Second Affiliated Hospital of Guangzhou University of Chinese Medicine, Guangzhou, China; ^6^ Guangdong Provincial Key Laboratory of Clinical Research on Traditional Chinese Medicine Syndrome, Guangzhou, China

**Keywords:** disease-modifying antirheumatic drugs (DMARDs), traditional Chinese medicine (TCM), DMARDs-like effects, rheumatic diseases, new strategies

Advances in antirheumatic drugs have brought about significant improvements for patients with rheumatic diseases, including rheumatoid arthritis (RA), systemic lupus erythematosus (SLE), and psoriatic arthritis (PsA). To achieve low disease activity or remission, disease-modifying antirheumatic drugs (DMARDs) remain unchanged as the primary line of treatment for the time being, providing improvements in signs and symptoms, physical function, social and work capacity, quality of life, as well as structural damage of cartilage and bone (Smolen et al., 2020). Currently, three categories of DMARDs are recommended for the management of these diseases, namely, conventional synthetic (cs) DMARDs (methotrexate (MTX), leflunomide, and sulfasalazine), biologic (b) DMARDs (antibodies against TNF-alpha (adalimumab, etanercept, golimumab, and infliximab), TNF-R, IL-6, and IL6-R), and targeted synthetic (ts) DMARDs [Janus kinase (JAK) inhibitors (tofacitinib, baricitinib, filgotinib and upadacitinib)] (Smolen et al., 2020). However, there is still a considerable number of patients who are non-responders to all DMARDs strategies. Moreover, these drugs are associated with adverse events such as severe infections, cardiovascular accidents events, malignancy, and gastrointestinal toxicity (Singh et al., 2015; de La Forest et al., 2017; Singh et al., 2020; Solomon et al., 2020). The economic burden of DMARDs is also non-negligible. These are the unmet needs for clinical care, turning patient treatments, turning our attention to exploring new sources for satisfactory antirheumatic drugs.

The worldwide prevalence of RA is 0.5%–1%, with the peak incidence usually between the ages of 40 and 50. Seronegativity, not meeting diagnostic criteria, or positive family history increase the population of RA approximately two to five times (Smolen et al., 2016; Cush, 2021). The increasing incidence of RA is driving growth of the global RA drugs market, in addition to increasing the awareness of RA among patients (Finckh et al., 2022). As a result, more RA drugs (innovative biologics, etc.) will be adopted and developed to drive the RA drugs market forward. Biosimilars are the fastest growing area because of their increased availability, low prices, and effectiveness. In the coming years, newer, more effective drugs are expected to bring about a dramatic shift in the RA drug market as well as in RA patients.

Historically, traditional Chinese medicine (TCM), which includes available isolated compounds, extracts, and herb formulas, has been utilized to treat different rheumatic diseases in China. Interestingly, many TCMs have good and definite DMARDs-like effects, as well as fewer side effects. They are less costly and can target multiple disease pathways compared with current DMARDs therapy, making TCMs a precious and valuable source for new anti-rheumatic drug development (Zhang et al., 2010; Wang et al., 2021). However, the clinical efficacy and mechanisms of TCM should be investigated and explored, and future international prospects and directions should be considered. In this Research Topic we highlight some clinical and experimental studies aimed at providing new strategies and insights into the treatment of rheumatic diseases using TCM.

In modern clinical practice, with highly performing densities in RA patients, plentiful TCM herbs and compounds have shown their potency and advantages in treating rheumatic diseases, with DMARDs-like properties (Lu et al., 2019; Li and Zhang, 2020; Wang et al., 2021). For instance, in the study of Chen et al. in our Research Topic, the total glucosides of paeony (TGP), which originates from Bai Shao (*Paeonia lactiflora* Pall.), can reduce disease activity and the disease recurrence rate in systemic lupus erythematosus (SLE), while reducing the incidence of adverse effects. A population-based retrospective cohort study performed by Chang et al. has shown that, in addition to conventional treatment, the cumulative incidence of RA was significantly lower in asthma patients treated with TCM, and that nine TCM formulas, including Xiao Qing Long Tang, were also associated with a lower risk of RA. Another renowned example, Sinomenine, extracted from the herb Qing Feng Teng [stem of *Sinomenium acuturn* (Thunb.) Rehd.et Wils and *Sinamenium acutum* (Thunb.) Rehd.et Wils.var.cinereum Rehd.et Wils.], which has been administered to rheumatic patients orally or through local injection for a long time, reduces pain, ameliorates morning stiffness, and improves both ultrasonographic blood flow and synovial thickness (Ding et al., 2022). Moreover, Lei Gong Teng (*Tripterygium wilfordii* Hook. f.), a novel antirheumatic drug source derived from TCM, which is widely used for RA treatment in Southeast Asia, provides good efficacy in response in combination therapy. The current challenge for this drug is to seek a viable way to reduce its toxicity towards human organs and systems (Wang et al., 2018; Zhou et al., 2018).

Mechanistically, the accumulating studies on TCM have shown that the multi-component, multi-target, and multi-path therapeutic approach of using TCM for complex diseases, such as rheumatic diseases, complements the single target or single disease shortcomings of DMARDs (Hopkins, 2008; Zhou et al., 2019; Wang et al., 2021). In the current Research Topic, dihydroartemisinin (DHA), extracted from the traditional Chinese herb *Artemisia annua* L., was found for the first time to alleviate bone destruction and joint oedema in a collagen-induced arthritis model, inhibiting B cells by activating the FcγRIIb/Lyn/SHP-1 signaling pathway (Hu et al.). Meanwhile, the diverse mechanisms of DHA were investigated by the same group in another study. NOD-like receptor protein (NLRP) 3 expression is inhibited by DHA through the hypoxia-inducible factor (HIF)-1α and Janus kinase (JAK) 3/signal transducer and activator of transcription (STAT) 3 signaling pathways (Zhang et al.). Interestingly, Li et al. (2006) showed that DHA can alleviate the symptoms of SLE-related nephritis by targeting the nuclear factor (NF)-κB signaling pathway to downregulate TNF-α secretion in BXSB mice. These studies not only demonstrate that DHA is a potential therapeutic drug, but also provide evidence that DHA and even TCM can treat rheumatic diseases through multiple targets and pathways. Another remarkable formulation research in this Research Topic evaluated the therapeutic effect of Qinghao-Biejia herb hair (QB) in ApoE−/− mice using SLE combined with atherosclerosis; the HMGB1/TLR4 signaling pathway was found to be a target of QB. In addition, QB could inhibit IL-6 and IFN-γ in MRL/lpr mice and considerably improved pathological lesions in mice with lupus nephritis (Hong et al.). These studies explored remarkable advances in the mechanisms and versatility of TCM for the treatment of rheumatic diseases, providing evidence and insights for the development of new drugs.

Decreased physical function, quality of life, and shortened lifespan are almost universal in patients with rheumatic diseases, but the long-term prognosis has improved over the last 20 years. These improvements may be mainly due to early diagnosis, aggressive treatment, and the widespread use and expanded selection of DMARDs. However, new drugs still need to be developed to reduce drug side effects, relieve patients’ financial stress, and improve drug response.

TCM is a huge creative treasure that restores the balance of various physiological systems holistically, with fewer side effects, lower costs, and multiple targets, which offers more potential therapeutic advantages than single-target therapy. In addition, the development of drug combination therapy offers promising prospects for the clinical application of TCM, especially in rheumatic diseases. Regrettably, there are still some issues in the current research, for example, the lack of rigorously designed, large-sample, and multi-center studies in clinical research. Besides, more active ingredients should be isolated from anti-rheumatic TCM, and systematic and in-depth mechanistic studies should be conducted to eventually apply them in clinical practice. This will complement the family of DMARDs from the field of TCM. There is no doubt that TCM brings benefits to patients with rheumatic diseases and is becoming increasingly important. We expect that new therapeutic methods will be developed from TCM and more scientists will be attracted to explore its latent energy.
